# The Hospital Boom

**Published:** 1892-04-30

**Authors:** 


### THE HOSPITAL BOOM.

By a Hospital Secretary.

The proposal to put before the public the case for the metropolitan voluntary hospitals by means of a volume written in their behalf by Mr. Egmont Hake, with an introduction by Mr. Walter Besant, is about to bear fruit. The publication originally arranged for Easter and postponed by the advice of an eminent firm of publishers, is now about to take place, and it may be interesting and advantageous to state the circumstances which gave rise to the undertaking.

The proposal, which originated in an address by Mr. Burdett to a meeting of hospital secretaries, held at his invitation in November last, was the outcome of a general feeling among hospital workers that the beneficent institutions they represent are but poorly appreciated by society at large, and that the inadequacy of the support rendered them is due to the fact that it is afforded by a comparatively small section of the community. The proposition received the unanimous approval of a large gathering, and it was agreed—(1) That the volume should be written in the interest of all the hospitals, general and special, which receive grants from the Hospital Sunday Fund; (2) that no mention of any particular hospital should be made in the body of the book; (3) that a plain statement of the work done by each institution and its necessities should be set forth in one uniform way in an appendix; and (4) that the several hospitals should be at liberty to utilise the publication for their individual benefit as their authorities should see fit.

A Committee was appointed, which reported to a second general meeting, held in January, when its proceedings were ratified, and it was arranged that a copy of the report should be forwarded to the secretary of every hospital concerned.

The financial question, which naturally confronted the Committee at the outset of its labours, might have proved a serious difficulty, but it was solved by the generous undertaking on the part of the Scientific Press (Limited) to guarantee £500, to cover the whole expense of the publication. The Committee thought, and rightly, that the hospitals for whose benefit the effort is made should assist by each subscribing for a certain number of copies of the book, in order to dispose of them among their supporters, and, to facilitate this, advance prospectuses and order forms have been forwarded to the several hospitals. It has been suggested that such profits as arise from the sale of the book shall be paid over to the Lord Mayor, at the Mansion House, as President of the Hospital Sunday Fund, and that donors who desire to render help to hospital work as a whole, rather than to any one particular institution, shall be invited to send their gifts to the Lord Mayor, in order that they also may be added to the Sunday Fund Collection for the current year.

A notice of this unique and promising effort would be incomplete without a recognition of what appears to us to be its chief feature, viz., the agreement upon the part of the metropolitan voluntary hospitals to enter upon united action to promote the common cause. This is of good augury, and there can be no truer friends of our hospitals than those who have laboured to bring about this first great effort in unison. The names of the authors of the volume are a guarantee that the case will be presented with fitting force, and it now behoves everyone interested in maintaining the voluntary and philanthropic character of the great work our hospitals represent, to assist in bringing home to the heart of the community, by means of this book, all that society owes to the cause of the sick poor and to medical science.

We are asked to state that invitations are about to be issued to a general meeting of hospital secretaries who have co-operated and who receive grants from the Hospital Sunday Fund, which will take place at St. Thomas's Hospital at the end of next week, when the attendance of a representative from every hospital concerned in the publication is urged.

